# Inhibitory Effect of Muscone on Porcine Epidemic Diarrhea Virus In Vitro

**DOI:** 10.3390/v18101111

**Published:** 2026-10-08

**Authors:** Shuwen Guan, Yaoying Jian, Linzhi Zhang, Zongyang Lv, Ruiming Hu, Huansheng Wu, Huali Xie, Deping Song, Zhen Ding, Zheng Chen

**Affiliations:** 1 Department of Preventive Veterinary Medicine, College of Animal Science and Technology, Jiangxi Agricultural University, Nanchang 330045, Chinahuanswu@jxau.edu.cn (H.W.);; 2 Yushui District Bureau of Agriculture, Rural Affairs and Grain, Xinyu 338000, China; 3 Jiangxi Provincial Key Laboratory for Animal Health, Institute of Animal Population Health, College of Animal Science and Technology, Jiangxi Agricultural University, Nanchang 330045, China; 4 Jiangxi Engineering Research Center for Animal Health Products, College of Animal Science and Technology, Jiangxi Agricultural University, Nanchang 330045, China

**Keywords:** porcine epidemic diarrhea virus, muscone, viral entry, caveolin-1

## Abstract

Porcine epidemic diarrhea virus (PEDV) is a major pathogen affecting the global swine industry, causing severe diarrhea, dehydration, and high mortality in neonatal piglets. Due to the continuous emergence of viral variants, the protective efficacy of currently available vaccines remains suboptimal, highlighting the urgent need for effective antiviral agents. In this study, we investigated the anti-PEDV activity of muscone in vitro. Our results demonstrated that muscone inhibits PEDV replication in a dose-dependent manner and exerts its antiviral effect primarily during the early stages of viral infection. Further mechanistic studies revealed that muscone acts mainly by interfering with viral entry into host cells. Specifically, Western blot analysis and confocal microscopy showed that muscone treatment led to the degradation of caveolin-1, suggesting that muscone may block PEDV entry by disrupting caveolin-mediated endocytosis. Collectively, our findings provide clear evidence that muscone possesses potent anti-PEDV activity in vitro and offer mechanistic insights into its mode of action, thereby establishing muscone as a promising lead compound for the development of novel antiviral strategies against PEDV.

## 1. Introduction

Porcine epidemic diarrhea virus (PEDV), a member of the genus *Alphacoronavirus* within the family *Coronaviridae*, is an enveloped, single-stranded, positive-sense RNA virus [1,2]. Its genome is approximately 28 kb in length and contains seven open reading frames (ORFs), encoding four structural proteins (spike protein S, envelope protein E, membrane protein M, and nucleocapsid protein N), sixteen non-structural proteins (nsp1–nsp16), and one accessory protein ORF3 [2,3]. Porcine epidemic diarrhea (PED), caused by PEDV, is an acute, highly contagious enteric disease that poses a particularly severe threat to neonatal piglets, manifesting as watery diarrhea, vomiting, and profound dehydration, with extremely high mortality [4,5]. Owing to its short incubation period and rapid transmission, the disease can affect an entire herd within days upon introduction into a farm, resulting in substantial economic losses to the global swine industry [6,7]. In 2010, highly pathogenic PEDV variants triggered large-scale outbreaks in southern China, causing the death of millions of piglets; subsequently, these variants spread swiftly to the United States, Canada, Mexico, and numerous Asian and European countries, giving rise to a global PED pandemic [8,9]. At present, PEDV has become one of the most devastating viral diseases threatening the Chinese swine industry.

Vaccination remains the primary control strategy against PED; however, the high mutation rate of the virus drives continuous antigenic variation, frequently compromising the protective efficacy of conventional vaccines [6,10,11]. The PEDV S protein serves as the main target of host immune responses, and its encoding gene is the most variable region in the viral genome, particularly the neutralizing antibody epitope domains within the S1 subunit, where mutations can enable immune escape from vaccine-induced immunity [12,13]. Vaccines developed based on the classical strain CV777 exhibit limited cross-protection against the currently prevalent G2-type variants, and single immunization provides only partial protection, insufficient to completely block viral infection and transmission [14]. Studies have shown that cross-protection between G2a and G2b subtypes of PEDV is low, further complicating the development of broad-spectrum vaccines [15]. Moreover, although inactivated vaccines are relatively safe, they fail to effectively elicit intestinal mucosal immune responses; even high levels of serum IgG in sows cannot provide adequate passive immunity to piglets via colostrum [10,16]. Live attenuated vaccines, while better mimicking natural infection and inducing intestinal IgA responses, carry the risk of reversion to virulence or recombination with field strains to generate novel variants [16,17]. Therefore, there is an urgent need to develop safe and effective anti-PEDV agents to compensate for the limitations of vaccination and reduce disease-associated losses.

Viral infections are often accompanied by robust host inflammatory responses, and PEDV infection can likewise trigger excessive apoptosis of intestinal epithelial cells and a cascade release of pro-inflammatory cytokines, accelerating the fatal outcome in piglets [18]. Muscone (3-methylcyclopentadecanone) is the major active component of the traditional Chinese medicine musk [19]. Modern pharmacological studies have demonstrated that muscone possesses excellent anti-inflammatory, antioxidant, and immunomodulatory properties. It effectively downregulates the activation of inflammatory pathways such as NF-κB, thereby alleviating tissue inflammatory damage [20,21]. Specifically, muscone significantly reduces the levels of lipopolysaccharide-induced inflammatory cytokines, including IL-1β, TNF-α, and IL-6 [22]. Following myocardial infarction, it attenuates cardiac macrophage-mediated inflammation by suppressing NF-κB and NLRP3 inflammasome activation, thus improving cardiac function [20]. In addition, muscone can exert anti-apoptotic activity by enhancing stress granule formation in acute ischemic stroke [23]. Although muscone has a well-established pharmacological basis for alleviating inflammation and protecting cells from stress-induced damage, whether it possesses direct anti-PEDV activity has not been reported to date.

Given the potential advantage of dual antiviral and anti-inflammatory intervention in the treatment of severe viral infections, this study selected muscone as the research subject. We aim to systematically evaluate its in vitro anti-PEDV activity, screen for effective inhibitory components, clarify the stage of action of the active compound, and preliminarily explore the underlying molecular mechanisms. This work is expected to provide new candidate compounds and reference targets for the development of anti-PEDV therapeutics.

## 2. Materials and Methods

### 2.1. Cells, Viruses, and Reagents

The African green monkey kidney (Vero-81) cell line (a highly permissive cell line to study coronaviridae infection) used in this study was maintained in our laboratory and routinely cultured in DMEM supplemented with 10% fetal bovine serum. A chimeric PEDV strain carrying the enhanced green fluorescent protein (EGFP) gene (GenBank accession No. JQ02316.1) was kindly provided by Dr. Zhen Li of the Shanghai Veterinary Research Institute, Chinese Academy of Agricultural Sciences. The wild-type PEDV strain (CH/JXJA/2017) was isolated from diarrheic pigs on a farm [22], propagated in Vero-81 cells, and titrated before use in subsequent experiments. The anti-PEDV N protein antibody was prepared in our laboratory. Muscone was purchased from Selleck Biotechnology Co., Ltd. (Houston, TX, USA) (Purity: 98.59%). It was dissolved in dimethyl sulfoxide (DMSO) as a stock solution, and the final concentration of DMSO in the culture system was ensured to have no cytotoxic effects in all experiments.

### 2.2. Cytotoxicity Assay

The cytotoxic effect of the compound on Vero-81 cells was evaluated using the CCK-8 assay. Vero-81 cells in the logarithmic growth phase were seeded into 96-well plates. When the cell density reached approximately 80%, the culture medium was replaced with fresh medium containing various concentrations (2 µM, 10 µM, 20 µM, and 50 µM) of the compound. After incubation for 24 h, the CCK-8 reagent was added according to the manufacturer’s instructions. Following an appropriate incubation period, the absorbance was measured at 450 nm using a microplate reader. Cell viability was calculated, and the data were processed with GraphPad Prism (v9.0) software to determine the safe concentration range of the compound for subsequent experiments.

### 2.3. Immunofluorescence Assay

Vero-81 cells were seeded into 12-well plates. After cell adherence, the cells were infected with EGFP-PEDV at a multiplicity of infection (MOI) of 0.05 for 2 h, followed by the addition of the compound. At 24 h post-infection, the distribution of EGFP-PEDV fluorescence was observed under a fluorescence microscope to evaluate viral replication.

### 2.4. Reverse Transcription Quantitative Real-Time PCR (RT-qPCR)

Vero-81 cells were seeded into 12-well plates. After cell adherence, the cells were infected with EGFP-PEDV at a multiplicity of infection (MOI) of 0.05 for 2 h, followed by the addition of the compound. At 24 h post-infection, total RNA was extracted from the cells, with uninfected Vero-81 cells serving as the control. RNA was reverse-transcribed into cDNA using Hifair III reverse transcriptase (Yeasen, Shanghai, China) according to the manufacturer’s instructions. Quantitative PCR was performed using the Hieff qPCR SYBR Green Master Mix (Yeasen) and specific primers, as described in our previous study [24]. Data were normalized to the expression level of GAPDH in each sample as an internal reference, and the quantitative differences between the control and treatment groups were determined using the 2^−ΔΔCt^ method. Each mRNA sample was assayed in triplicate, and the results were statistically analyzed using GraphPad Prism 10 software.

### 2.5. Virus Titration by TCID_50_ Assay

Vero-81 cells were seeded into 12-well plates. When the cell density reached approximately 80%, the cells were incubated with increasing concentrations of muscone (2 µM, 10 µM, 20 µM, and 50 µM) for 1 h, washed with PBS, and then infected with PEDV at an MOI of 0.05 for 2 h. The cells were subsequently cultured in virus maintenance medium containing the corresponding concentrations of muscone for 24 h. After incubation, the supernatants were collected for TCID_50_ determination to assess viral titers. Briefly, ten-fold serial dilutions (from 10^−1^ to 10^−10^) of the supernatant samples were added to Vero-81 cells seeded in 96-well plates, with eight replicate wells per dilution. Following a 3-day incubation, the cytopathic effect (CPE) was observed under light microscopy, and the TCID_50_ values were calculated using the Reed–Muench method.

### 2.6. Western Blot Analysis

Vero-81 cells were lysed using RIPA lysis buffer containing 10 mM phenylmethylsulfonyl fluoride (PMSF) to obtain protein lysates. Equal amounts of protein samples were separated by 10% sodium dodecyl sulfate-polyacrylamide gel electrophoresis (SDS-PAGE) and then transferred onto polyvinylidene difluoride (PVDF) membranes. The membranes were blocked with 5% bovine serum albumin (BSA) or non-fat milk and then incubated separately with anti-β-actin and anti-PEDV N protein polyclonal antibodies at 4 °C overnight. After three washes with TBST, the membranes were incubated with horseradish peroxidase (HRP)-conjugated secondary antibodies for 1 h at room temperature, followed by three additional washes with TBST. Protein bands were visualized using ECL reagent (Yeasen) with the Amersham ImageQuant 800 system (GE Healthcare, Waukesha, WI, USA).

### 2.7. Time-of-Addition Assay

Vero-81 cells in the logarithmic growth phase were seeded into 12-well plates. When the cell density reached approximately 80%, muscone was added to the cells at different time points: 2 h before viral infection, simultaneously with viral infection, and 2 h post-infection. At 24 h post-infection, the supernatants were discarded, RNA extraction reagent was added, and total RNA was extracted for RT-qPCR analysis.

### 2.8. Virus Attachment and Entry Assays

Attachment assay: Vero-81 cells were seeded into 12-well plates and cultured to approximately 80% confluence. The cells were then incubated with a mixture of PEDV (MOI = 0.05) and the compound at 4 °C for 1 h. Cells infected with PEDV in the presence of the same concentration of DMSO served as the control. After washing with PBS, total RNA was collected, and the level of viral RNA in the cells was quantified by RT-qPCR.

Entry assay: Vero-81 cells were seeded into 12-well plates and cultured to approximately 80% confluence. The cells were first incubated with PEDV (MOI = 0.05) at 4 °C for 1 h to allow viral attachment. Unbound viruses were removed by washing, and then the compound was added. The cells were subsequently shifted to 37 °C and incubated for 2 h to permit viral entry. Cells infected with PEDV in the presence of the same concentration of DMSO served as the control. After washing with PBS, total RNA was collected, and the level of viral RNA in the cells was measured by RT-qPCR.

### 2.9. Virucidal Assay

PEDV was incubated with various concentrations of muscone at 37 °C for 2 h. The mixture was then added to Vero-81 cells and incubated at 37 °C for 2 h, after which unbound viruses were removed by washing with PBS. Cells infected with the same amount of PEDV without compound treatment served as the control group. The cells were cultured at 37 °C until more than 80% cytopathic effect (CPE) was observed, and the supernatants were collected after three freeze–thaw cycles for subsequent detection. Viral inactivation was then evaluated by TCID_50_ assay and compared with the control group infected with the same amount of virus but without compound treatment.

### 2.10. Statistical Analysis

All experiments were independently repeated at least three times. Data are presented as the mean ± standard deviation (SD). Statistical analysis was performed using GraphPad Prism 9.0 software. Differences were considered statistically significant at * *p* < 0.05, ** *p* < 0.01, and *** *p* < 0.001; ns denotes no significant difference.

## 3. Results

### 3.1. Cytotoxicity Evaluation of Muscone

**Figure 1:**
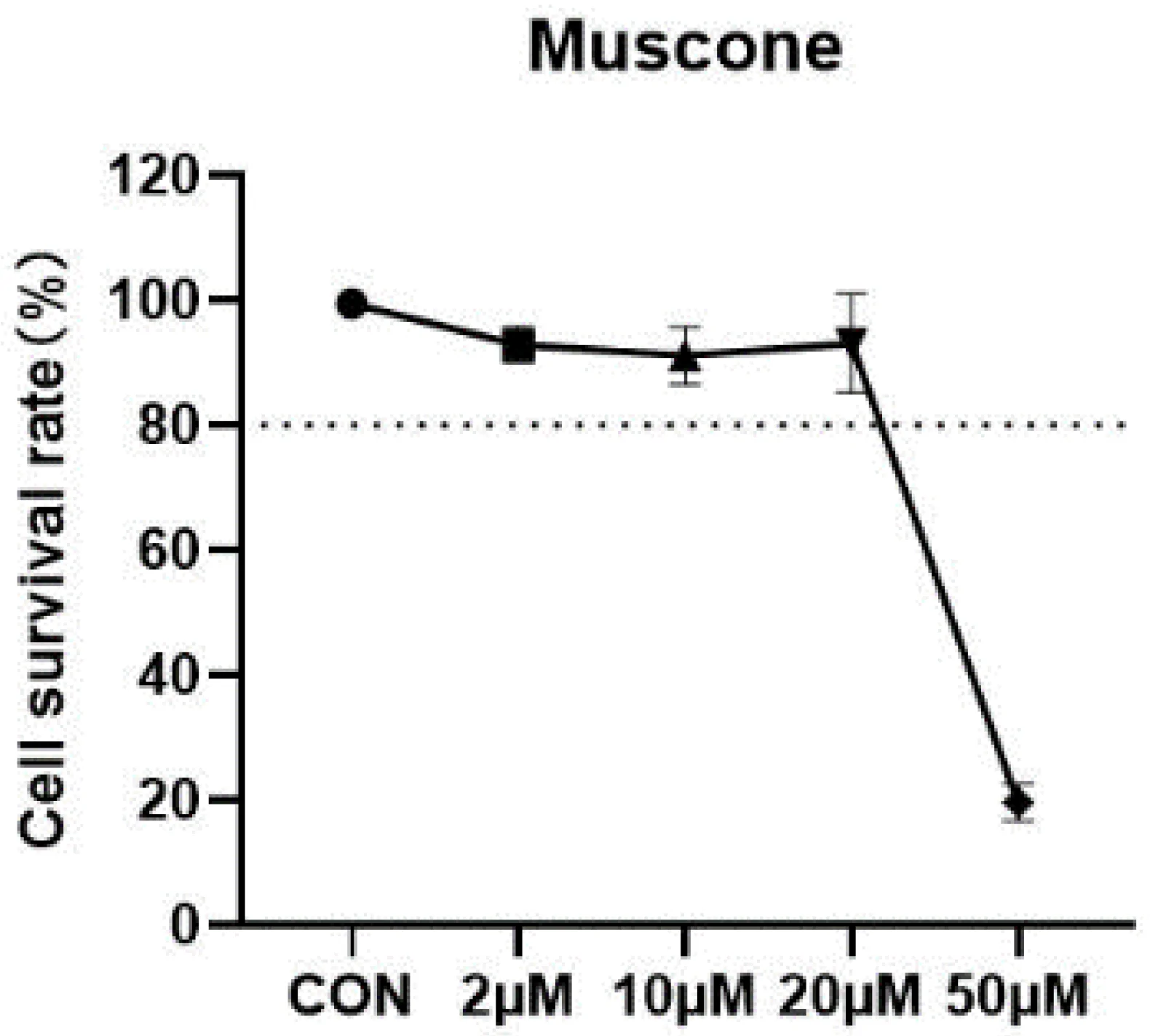
Cell viability of muscone-treated cells assessed by CCK-8 assay. Vero-81 cells were treated with various concentrations (0 μM, 2 μM, 10 μM, 20 μM and 50 μM) of muscone. After 24 h, cell viability was measured using the CCK-8 kit according to the manufacturer’s instructions.

Many drugs are inherently cytotoxic, and the solvent DMSO can also introduce additional toxicity. To assess the cytotoxicity of muscone against VERO-81 cells, we performed a CCK-8 assay according to our previous study. As shown in Figure 1, the results showed that muscone exhibited no obvious cytotoxicity at concentrations up to 20 µM, with cell viability remaining above 80%. This safe concentration range was subsequently used for the evaluation of antiviral activity.

### 3.2. Dose-Dependent Inhibition of PEDV Replication by Muscone

**Figure 2:**
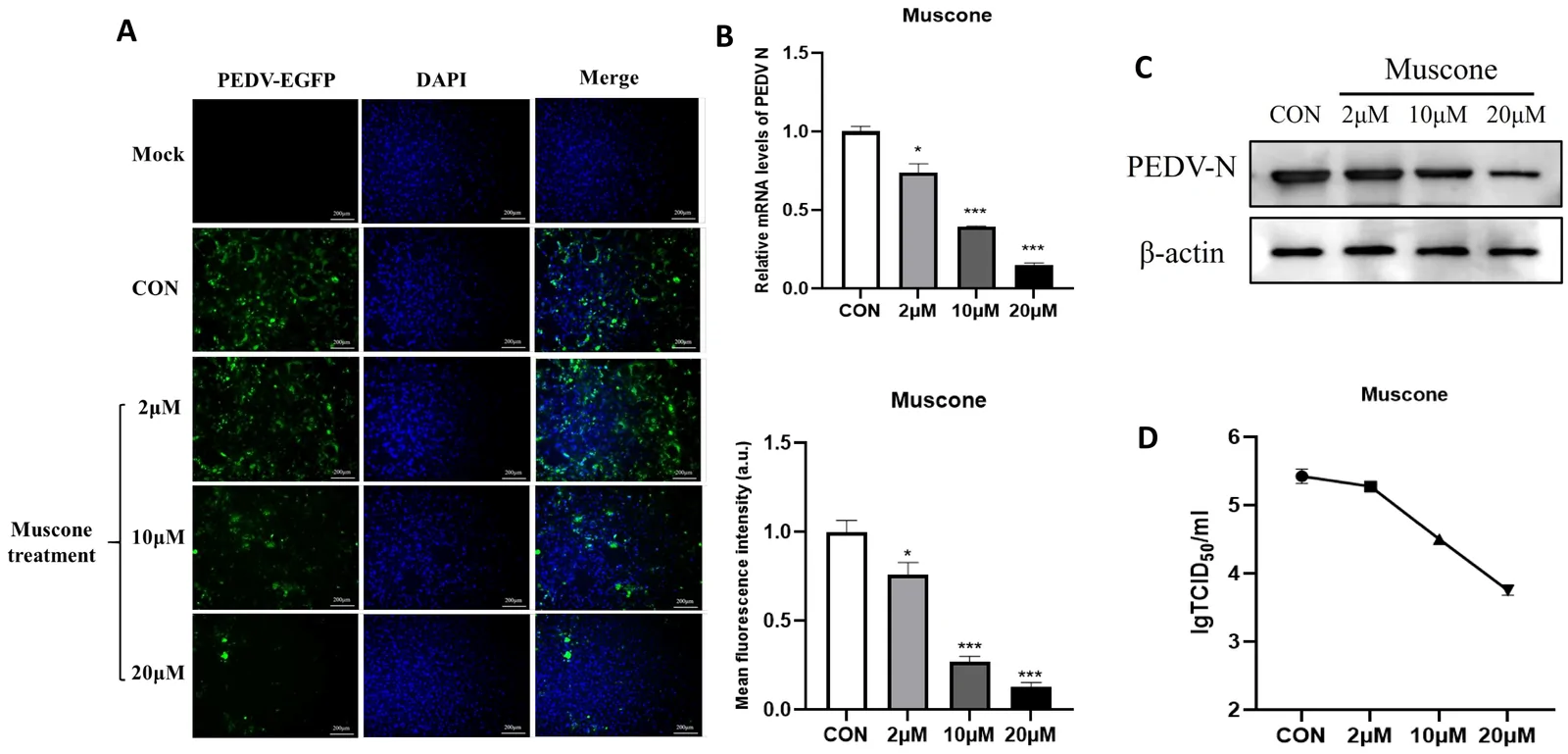
Dose-dependent inhibition of PEDV replication by muscone. Vero-81 cells were pretreated with muscone (2, 10, or 20 µM) for 1 h, then infected with PEDV for 2 h. After infection, the cells were cultured in virus maintenance medium supplemented with the same concentrations of muscone for 24 h. (**A**) The fluorescence signals were visualized under a fluorescence microscope. (**B**) Cell samples were harvested, and PEDV mRNA levels were quantified by RT-qPCR. (**C**) Cell lysates were harvested and subjected to Western blot analysis using a PEDV N monoclonal antibody. (**D**) Cell supernatants were harvested, and PEDV titers were determined by the TCID_50_ assay. The data presented are the results of three independent experiments. *, *p* < 0.05; ***, *p* < 0.001.

To further confirm the antiviral effect of muscone and evaluate its dose-dependent activity against PEDV, Vero-81 cells were incubated with increasing concentrations of muscone (2, 10, and 20 µM) prior to infection. After 24 h of infection, a portion of cells was subjected to fluorescence microscopy for observation of fluorescence intensity, while the remaining cells were harvested as pellets, along with the culture supernatants, and then subjected to qPCR, Western blotting, and TCID_50_ assays. The results demonstrated that increasing concentrations of muscone led to a dose-dependent reduction in EGFP fluorescence intensity (Figure 2A), PEDV N gene mRNA levels (Figure 2B), N protein expression (Figure 2C), and progeny viral titers (Figure 2D). Additionally, using the above data, we calculated that the IC_50_ of muscone is 8.22 µM and that the selectivity index is 4.30, as shown in Table 1. These findings confirm that muscone effectively inhibits PEDV replication in vitro, with its inhibitory potency positively correlated with the administered concentration.

### 3.3. Time-Dependent Effect of Muscone on PEDV Replication

**Figure 3:**
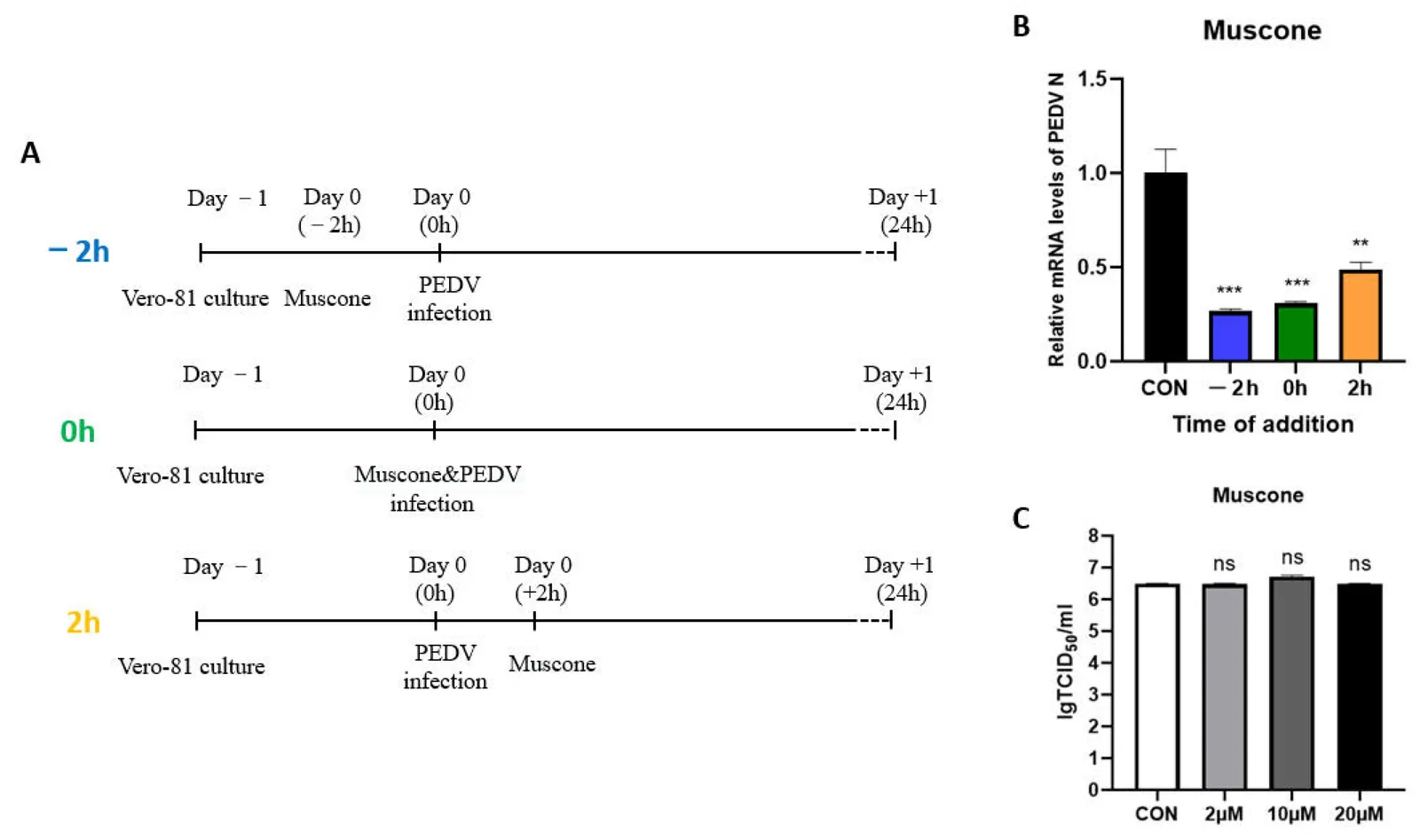
Time-of-addition and direct virucidal effects of muscone against PEDV. (**A**) Schematic diagram of Muscone processing time. (**B**) Cells were treated with muscone as indicated in the schematic, and viral replication was assessed by RT-qPCR to evaluate the effect of different addition regimens. CON denotes the DMSO-treated control. (**C**) Vero-81 cells were inoculated with virus that had been pre-incubated with muscone (2, 10, or 20 µM) at 37 °C for 2 h. Following 2 h of adsorption, the cells were maintained until approximately 80% of the monolayer showed detachment. The culture supernatants were collected, freeze-thawed three times, and titrated by the TCID_50_ method according to Reed–Muench. Results are from three independent experiments. **, *p* < 0.01; ***, *p* < 0.001; ns, not significant.

To identify the specific stage of the PEDV life cycle targeted by muscone, the compound was added at different time points relative to infection—before, during, and after viral inoculation—and viral replication levels were subsequently measured, as illustrated in the schematic diagram (Figure 3A). The most pronounced inhibition was observed when muscone was administered either before (−2 h) or simultaneously with (0 h) infection; however, the inhibitory effect was markedly diminished when treatment was delayed until after viral attachment (2 h) (Figure 3B). Furthermore, a virucidal assay demonstrated that pre-incubation of muscone with free virions in vitro did not reduce viral infectivity (Figure 3C), indicating that muscone lacks direct virus-killing activity. Collectively, these findings suggest that the antiviral action of muscone does not target the viral particles themselves but rather acts at an early stage of the viral infection cycle.

### 3.4. Muscone Blocks PEDV Entry into Cells

**Figure 4:**
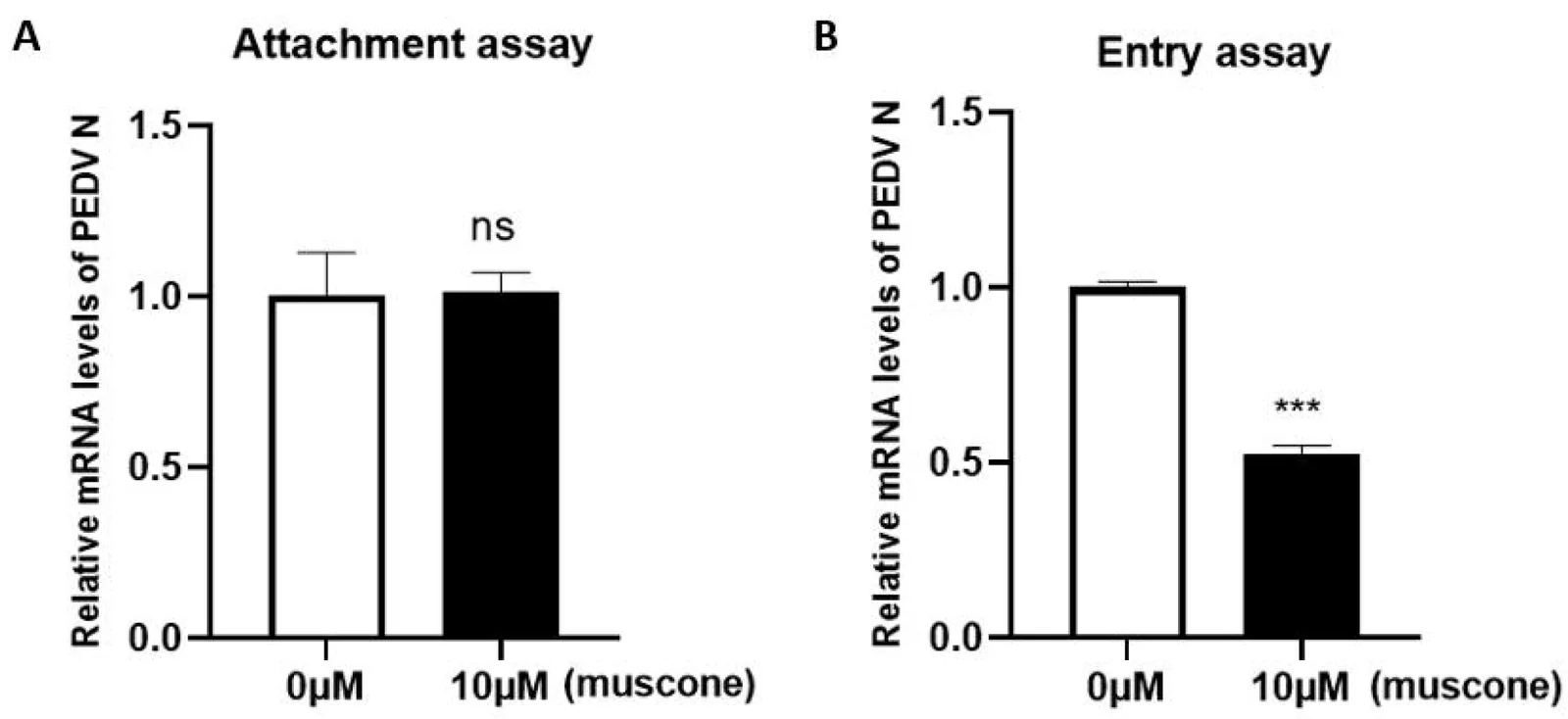
Muscone inhibits PEDV entry but does not affect viral attachment. Viral RNA levels in cells treated with muscone at a concentration of 10 µM during either the viral attachment phase (**A**) or the entry phase (**B**) were measured using qPCR. Results are from three independent experiments. ***, *p* < 0.001; ns, not significant.

Previous experiments revealed that muscone primarily acts at the early stage of viral infection. To further explore in which step of the PEDV life cycle muscone functions, qRT-PCR was applied to evaluate the effect of the compound on PEDV attachment and entry. At 4 °C, a condition that permits viral attachment to the cell surface but blocks endocytosis, treatment with muscone did not significantly affect the viral mRNA levels (Figure 4A), indicating that the binding of viral particles to cells was not substantially influenced. However, when the temperature was shifted to 37 °C to initiate the viral entry step, the mRNA expression level of PEDV N in muscone-treated cells was significantly reduced compared with that in the untreated control group (Figure 4B). These results demonstrate that muscone exerts its antiviral function by blocking the critical step of PEDV entry into host cells.

### 3.5. Muscone Inhibits PEDV Entry into Cells by Degrading Caveolin-1

**Figure 5:**
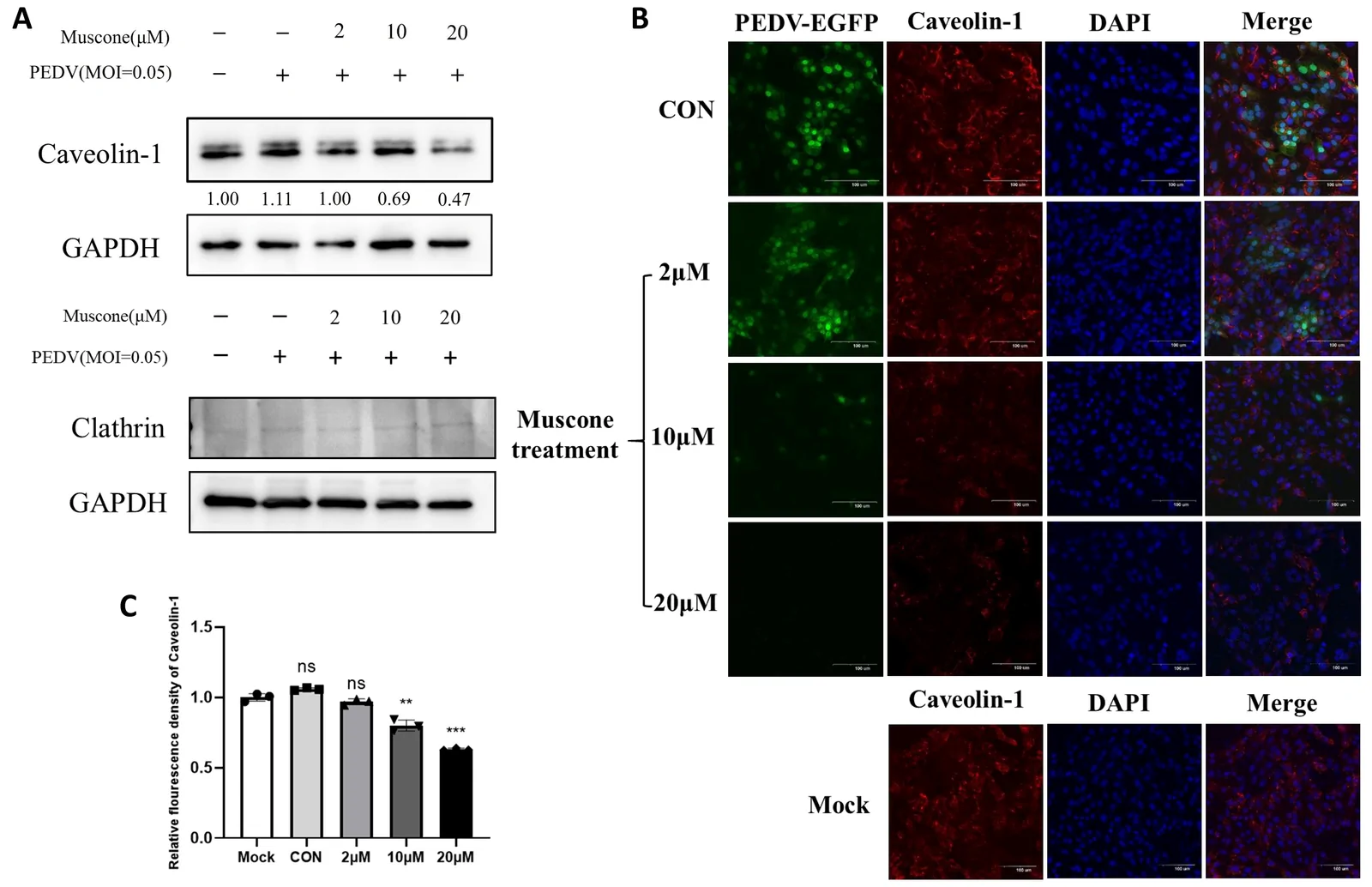
Muscone inhibits PEDV entry by inducing degradation of Caveolin-1. Vero-81 cells were pretreated with muscone (2, 10, or 20 µM) for 1 h, then infected with PEDV for 2 h. After infection, the cells were cultured in virus maintenance medium supplemented with the same concentrations of muscone for 24 h. (**A**) Cell lysates were harvested and subjected to Western blot analysis using Clathrin and Caveolin-1 polyclonal antibodies. (**B**) Cells were fixed with paraformaldehyde, stained with an anti-Caveolin-1 polyclonal antibody for immunofluorescence, and then examined under a confocal laser scanning microscope. (**C**) Fluorescence intensity was quantified by densitometric analysis. Results are from three independent experiments. **, *p* < 0.01; ***, *p* < 0.001; ns, not significant.

Previous studies have shown that PEDV enters host cells primarily via clathrin-mediated and caveolae-dependent endocytosis [25,26]. Our finding that muscone inhibits PEDV entry suggested that muscone might interfere with these endocytic pathways. To explore the underlying mechanism, we examined the expression levels of clathrin and caveolin-1 following muscone treatment. Western blot analysis revealed that muscone significantly reduced the protein level of caveolin-1 in a time- and dose-dependent manner, whereas the expression of clathrin, a key protein in another major endocytic route, remained unaffected (Figure 5A). This observation was further corroborated by confocal laser scanning microscopy, which showed a marked decrease in caveolin-1 fluorescence intensity after muscone treatment (Figure 5B,C). Collectively, these results indicate that muscone likely inhibits PEDV entry by inducing the degradation of caveolin-1, thereby disrupting caveolae-mediated endocytosis.

## 4. Discussion

Muscone, the principal bioactive component of musk, has been extensively characterized for its anti-inflammatory, antioxidant, and immunomodulatory properties [20,21,22,23]. However, whether muscone possesses direct antiviral activity against PEDV had remained unexplored prior to this study. Here, we demonstrate for the first time that muscone exerts a significant inhibitory effect on PEDV replication in vitro and further show that this antiviral activity is achieved by blocking viral entry via caveolin-1 degradation.

Our results revealed that muscone dose-dependently reduced EGFP-PEDV fluorescence intensity, PEDV N gene mRNA levels, N protein expression, and progeny viral titers, with no obvious cytotoxicity at concentrations up to 20 µM. This indicates that the observed antiviral effect reflects genuine inhibition of viral replication rather than non-specific host cell damage. More importantly, time-of-addition experiments showed that muscone was most effective when added before or simultaneously with infection, whereas its inhibitory effect was largely lost when administration was delayed until after viral attachment. In line with these findings, a virucidal assay confirmed that muscone did not inactivate free virions. Collectively, these results position the antiviral action of muscone at an early stage of the viral life cycle—specifically, the pre-entry or entry phase—rather than at the level of viral particle integrity.

Further mechanistic investigation pinpointed the viral entry step as the primary target. At 4 °C, a temperature that permits virus attachment but blocks endocytosis, muscone treatment did not significantly alter viral mRNA levels, suggesting that binding to the cell surface remained intact. However, upon shifting to 37 °C to allow entry, muscone markedly reduced PEDV N mRNA expression. This observation strongly indicates that muscone specifically interferes with the internalization process, without affecting initial attachment.

PEDV is known to enter host cells primarily via clathrin-mediated and caveolae-dependent endocytosis [25,26]. In agreement with this, our mechanistic data showed that muscone significantly reduced caveolin-1 protein levels in a time- and dose-dependent manner, as confirmed by both Western blotting and confocal microscopy, whereas clathrin expression remained unchanged. These results suggest that muscone selectively disrupts the caveolae-mediated endocytic pathway by inducing caveolin-1 degradation, thereby impairing PEDV entry—providing a plausible molecular explanation for its antiviral activity.

It is worth noting that host-targeted antiviral strategies are relatively uncommon among reported natural products with anti-PEDV activity. Most known natural products act directly on viral components, such as salvianolic acid A targeting the S protein [27], and ethyl caffeate or luteolin targeting the 3CL protease [28,29]. In contrast, muscone blocks viral entry by downregulating caveolin-1. This host-directed mechanism may offer a distinct advantage in the face of viral evolution, as targeting host factors is less prone to resistance conferred by mutations in viral genes.

Beyond its antiviral effect, muscone also exhibits well-established anti-inflammatory activity, including inhibition of NF-κB and NLRP3 inflammasome activation and suppression of IL-1β, TNF-α, and IL-6 production [19,20]. Given that PEDV infection triggers excessive apoptosis of intestinal epithelial cells and a pro-inflammatory cytokine cascade—key pathological drivers of piglet mortality—muscone may confer additional therapeutic benefit by alleviating intestinal damage through its anti-inflammatory action. For neonatal piglets, whose immune systems are not yet fully mature, this dual antiviral and anti-inflammatory profile could be more advantageous than single-mechanism agents.

Despite these promising in vitro findings, several limitations should be acknowledged. First, all experiments were performed in the Vero-81 cell line; whether muscone exhibits similar antiviral efficacy in porcine intestinal epithelial cells—the natural target cells of PEDV in vivo—remains to be determined. Nevertheless, Vero-81 cells are widely used for PEDV propagation and antiviral screening and can provide a reproducible system for comparing compounds under standardized conditions. Therefore, the lack of validation in natural target cells limits the physiological interpretation of the results but does not invalidate the initial screening findings. Future studies using porcine intestinal epithelial cells or intestinal organoids would help clarify whether the observed antiviral activity is maintained in a more relevant host cell context. Second, the in vivo efficacy and safety of muscone against PEDV have not been evaluated and are essential prerequisites for any potential therapeutic application. Third, the precise molecular machinery by which muscone induces caveolin-1 degradation—whether via the ubiquitin-proteasome pathway, autophagy-lysosomal system, or transcriptional/translational regulation—requires further elucidation.

In summary, this study demonstrates that muscone inhibits PEDV replication in vitro by blocking viral entry through caveolin-1 degradation, thereby disrupting caveolae-mediated endocytosis. To our knowledge, this is the first report of direct anti-PEDV activity for muscone. Given its combined antiviral and anti-inflammatory properties, muscone represents a promising lead compound for anti-PEDV drug development. Moreover, the identification of caveolin-1-mediated entry as a targetable pathway may provide valuable mechanistic insights for the design of host-directed antivirals against PEDV and related coronaviruses.

## Figures and Tables

**Table 1:** Selectivity index of muscone.

CC_50_ (μM)	IC_50_ (μM)	SI
35.36	8.22	4.30

## Data Availability

Data is contained within the article.

## Abbreviations

The following abbreviations are used in this manuscript:

EGFP	Enhanced green fluorescent protein
DMEM	Dulbecco’s Modified Eagle Medium
DMSO	Dimethyl sulfoxide
FBS	Fetal Bovine Serum
PBS	Phosphate-buffered saline
CCK8	Cell Counting Kit-8
CC50	50% Cytotoxic Concentration
IC50	50% Inhibitory Concentration
GAPDH	Glyceraldehyde-3-phosphate dehydrogenase
qRT-PCR	Quantitative real-time PCR
CPE	Cytopathic effect
TCID50	Median tissue culture infective dose
PMSF	Phenylmethylsulfonyl fluoride
SDS-PAGE	Sodium dodecyl sulfate-polyacrylamide gel electrophoresis
HRP	Horseradish peroxidase
MOI	Multiplicity of infection
PEDV	Porcine epidemic diarrhea virus
